# A Review on Latent Autoimmune Diabetes in Adults

**DOI:** 10.7759/cureus.47915

**Published:** 2023-10-29

**Authors:** Vijay Ravikumar, Ariba Ahmed, Ashish Anjankar

**Affiliations:** 1 Medical Education, Jawaharlal Nehru Medical College, Datta Meghe Institute of Higher Education & Research, Wardha, IND; 2 Biochemistry, Jawaharlal Nehru Medical College, Datta Meghe Institute of Higher Education & Research, Wardha, IND

**Keywords:** type 1 and type 2 diabetes mellitus, dpp-4 inhibitors, : metformin, intensive insulin therapy, latent autoimmune diabetes in adults (lada)

## Abstract

Latent autoimmune diabetes (LADA) is an unique form of diabetes that has characteristics of both type 1 and type 2 diabetes. Type 1.5 diabetes also known as LADA is occasionally confused for type 2 diabetes because there is delay in presenting features and early insulin independence. LADA, on the other hand, is an autoimmune disorder that differs from type 2 diabetes in that autoantibodies against pancreatic beta cells are what characterise it. Insulin production eventually diminishes due to the autoimmune destruction of pancreatic beta cells as a result of the pathophysiology of LADA. Autoantibodies to glutamic acid decarboxylase (GAD), islet antigen-2 (IA-2), and insulin are frequently detected in LADA patients. These autoantibodies have important implications for therapy strategies and are essential in differentiating LADA from type 2 diabetes. LADA clinical management is very challenging. The aim of this article is to view the characteristics, disease presentation, diagnostic challenges, progression and treatment modalities of LADA.

## Introduction and background

Diabetes, a critical threat to global public health, represents the ailment with the most rapid worldwide proliferation rate. Given its chronic nature, primary and secondary prevention play essential roles in mitigating the impact it imposes on individuals and society. To effectively initiate primary prevention, comprehending the factors influencing the onset of this condition becomes crucial. Diabetes, fundamentally, encompasses a range of illnesses, extending from the classic insulin-dependent type 1 diabetes to the prevalent insulin-resistant type 2 diabetes [1].

Diabetes mellitus (DM) arises when blood glucose levels fail to achieve proper control. Its classifications are type 1, type 2, maturity-onset diabetes of the young (MODY), gestational diabetes, neonatal diabetes, steroid-induced diabetes, and autoimmune diabetes [2]. The more common variant in the realm of adult autoimmune diabetes is known as latent autoimmune diabetes in adults (LADA), constituting 3% to 11% among the population [3].

Frequently referred to as type 1.5 diabetes, this condition exhibits phenotypic, genetic, and pathophysiological characteristics with both type 1 diabetes (T1DM) and type 2 diabetes (T2DM) [4]. T1DM is an autoimmune disorder necessitating insulin therapy since insulin-producing beta cells are lost. While it predominantly manifests in children, some individuals exhibit symptoms during adulthood. LADA is the term used to describe these individuals initially presumed to have T2DM [5].

Specific autoantibodies targeting pancreatic islet cells and the initial requirement for insulin treatment are two hallmarks of T1DM and LADA. The prevalence of this disorder is similar in adults as in children. Although initially not dependent on insulin, the presenting features of late-onset autoimmune diabetes are more akin to those with T2DM than T1DM, potentially leading to an inadvertent misdiagnosis. Many physicians and endocrinologists regard LADA and T1DM as closely related forms of autoimmune diabetes, and LADA is a unique subsistence and subject of discussion [6].

The diagnostic criteria and differential diagnosis for LADA remain somewhat unclear due to its latent and slowly progressing nature, which increases the risk of misdiagnosis [7]. The Immunology of Diabetes Society has established three crucial criteria for identifying LADA, including individuals over 30, any autoantibodies against islet cells, and the absence of insulin dependence for at least six months following diagnosis [8]. Nevertheless, there continues to be room for disagreement regarding diagnostic standards and the concept of LADA. Furthermore, selecting a standardized treatment protocol for LADA is challenging due to its substantial variability, necessitating personalized care [9].

Misdiagnosing LADA can significantly worsen patients' health and impose a substantial financial burden on society. Glutamic acid decarboxylase autoantibody (GADA)-negative individuals with a phenotypic T2DM representation and LADA patients face a relative risk of complications and mortality [10]. This review aims to underscore the similarities between LADA and T1DM explore potential etiological and pathophysiological aspects of LADA, summarize data indicating the involvement of environmental and genetic factors in the development of LADA, and present the current diagnostic criteria for LADA.

## Review

Methodology

**Figure 1:**
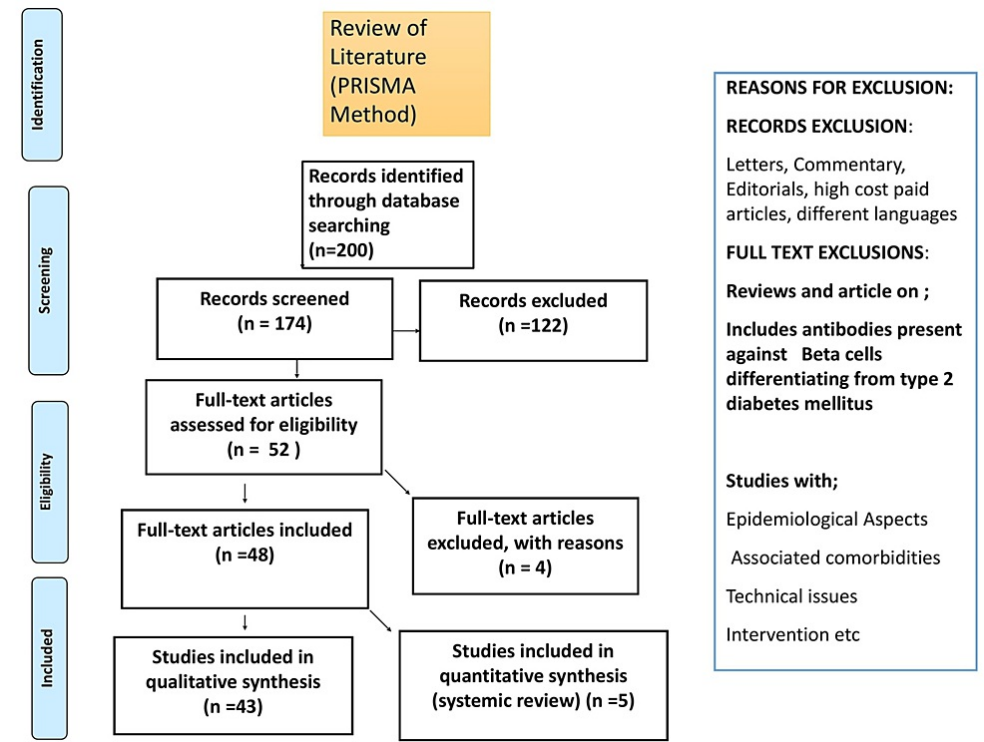
Literature search strategy by Preferred Reporting Items for Systematic Reviews and Meta-Analyses (PRISMA) method

The methodology employed a thorough literature search strategy utilizing multiple electronic databases, namely PubMed, Scopus, and Google Scholar. The search terms employed were relevant to "antidiabetic agents," "insulin therapy," "T1DM and T2DM," "dpp-4 inhibitors," and "Metformin." In addition to the electronic database searches, a manual search was conducted on the reference lists of pertinent articles and review papers to uncover additional studies. Language restrictions were not imposed; only studies published up to the current knowledge as of 2023 were considered. Included in the study were investigations into the potential of antidiabetic agents as therapeutic interventions for latent autoimmune diabetes in adults, as well as for T1DM and T2DM. This study encompassed a range of research types, including in vitro studies, animal models, and clinical studies with various designs. Exclusion criteria comprised studies exclusively focused on diabetes and review articles, editorials, commentaries, and conference abstracts. Two different reviewers first skimmed the titles and abstracts while carefully evaluating the complete contents of their chosen documents. Any discrepancies have been settled by consensus or, if required, by talking to a third reviewer. Concerning T2DM, the technique intended to include top-notch research contributing to a thorough understanding of the disease causes and treatment regimens. By implementing a rigorous search strategy and applying stringent inclusion and exclusion criteria, a robust selection of studies was identified to inform the review. The literature search strategy, adhering to the Preferred Reporting Items for Systematic Reviews and Meta-Analyses (PRISMA) method, is presented in Figure 1.

Epidemiology

According to epidemiological research, LADA may be responsible for 3-11% out of all occurrences of adult-onset diabetes. In people with T2DM living in Western nations, the incidence of LADA ranges from 2.91% to 10% [11]. According to statistics gathered from Italian registries, the frequency of T1DM in people between the ages of 29 and 50 is comparable to that of teenagers between the ages of 14 and 21 [12].

According to data published in LADA, over 37% of T1DM cases include individuals above 29. The actual frequency of this disorder among adults between the ages of 14 and 36 is much times greater than previously thought [13]. According to the Diabetes Outcomes Progression Trial, the prevalence of GAD autoantibody positivity among people with T2DM is 3.82% in Europe and 4.11% in North America. Autoantibodies were reported in 17 of 499 (3.4%) T2DM patients, of whom 12 were GAD65 positive and seven were islet antigen-2 (IA-2) positive, with one subject testing positive for both. This study revealed that islet cell autoimmunity was much less prevalent in T2DM [14]. The frequency appears to vary among various communities and nations, potentially due to differences in research design, inclusion standards, and lifestyle differences [15].

Pathogenesis

T1DM, T2DM, and LADA overlap among the broader ballpark of elderly-onset diabetes without any particular distinctions [9]. The continuous line-based distribution of pathogenic variables, such as immunological activity, insulin resistance and beta-cell function, can distinguish between different types of diabetes mellitus [16]. Since cases of typical T1DM and adult-onset autoimmune diabetes are challenging to differentiate immunologically from each other, young-onset T1DM has a significant immunogenetic load and a more rapid impairment of beta-cells as evidenced by lower C-peptide levels and a quicker fall in C-peptide levels [17].

LADA is characterized by the same diabetes-associated autoantibody (DAW) as traditional T1DM. GADAs are considered sensitive indicators of T2DM and adult-onset T1DM [18]. At the time of diagnosis, LADA patients release more insulin than T1DM patients, which implies that the pathophysiology may include processes other than the autoimmune destruction of beta-cells. It has been established that LADA patients experience insulin resistance as a result [19].

LADA appears to be at the opposite end of the autoimmune diabetes spectrum from classical T1DM, where a combination of genetic susceptibility, an autoimmune response, and the manifestation of the non-insulin-necessity creates an autoimmune diabetes with pathogenic characteristics but a moderate type more akin to T2DM [9].

Genetic causes

Studies have indicated that individuals with LADA share genetic traits with T1DM and T2DM. Research by Pan et al. and Zhou et al. underscores the importance of prioritizing susceptible genes in diagnosing LADA [20]. The genes within the human leukocyte antigen (HLA) complex account for almost half of the genetic predisposition and have the most substantial genetic influence on autoimmune diabetes [21].

Conversely, the association of hereditary threat with T2DM seems influenced by many similar genetic variations, contributing only a fraction to the hereditary predisposition to the disease [22]. In LADA, there is a lower prevalence of specific HLA alleles, specifically HLA-DRB104-DQB10302 and HLA-DRB10301-DQB10201, which are highly prevalent in young-onset T1DM and become less frequent in older people. Similar trends have been seen with other genotypes also [23].

Furthermore, LADA exhibits a strong association with the T2DM-associated variant transcription factor 7-like 2 (TCF7L2), particularly in cases involving obesity. HLA, INS, and PTPN22 have been linked to genetic resemblances between T1DM and T2DM. These findings support the notion that LADA represents a blend of characteristics from T1DM and T2DM, suggesting that individuals with LADA may share hereditary features of both types of diabetes mellitus [24]. In patients with diabetes-related autoantibodies, gene risk scores can aid in stratifying progression rates to insulin dependence and identifying cases likely to exhibit false positive autoantibodies [25].

Diagnostic criteria

In the existing literature, the age spectrum for diagnosis of LADA varies between 15 and 45 years old, depending on geographical location. In the United Kingdom Prospective Diabetes Study, the age parameters for including LADA patients spanned from 25 to 65 years of age [26]. According to findings from the Diamond Study, a distinction between juveniles and grown-ups is made at 15. Consequently, expanding the age threshold could facilitate physicians in screening a broader cohort for LADA, ultimately leading to swifter diagnosis and treatment of this condition [7].

In theory, it is advisable to conduct tests for islet autoantibodies in all instances of recently diagnosed T2DM to pinpoint cases affected due to LADA. Detecting LADA early and instituting a tailored therapeutic approach may prove pivotal in averting autoimmune progression and conserving beta-cell functionality [6]. The presence of antibodies within the bloodstream plays a pivotal role in identifying LADA. Five serum autoantibodies are indicative of humoral immunity in LADA: GADA, pancreatic islet-cell antibodies (ICA), autoantibodies against insulin (IAA), insulinoma associated-2 autoantibodies (IA-2A), and association of zinc transporter-8 autoantibody (ZnT8A) [15].

Among these, GADA is acknowledged as one of the most sensitive immunological criteria for diagnosing LADA, owing to its manifestation in the early stages and its prolonged presence in the bloodstream. Notably, the GADA assay boasts the highest degree of standardization among all autoantibody tests. Furthermore, individuals with low GADA titers are categorized as subjects with T2DM and metabolic syndrome, whereas those with elevated GADA titers display resemblances to T1DM patients [27].

LADA diagnostic criteria also encompass elements related to beta-cell function and chronic complications. LADA typically commences as T2DM without insulin dependency, subsequently evolving towards beta-cell apoptosis, mirroring the initial stages of T2DM with insulin independence. Intriguingly, there is a decline of functioning beta cells in these patients that surpasses that of individuals with T2DM by a factor of three. Identifying diabetic patients with a heightened probability of having LADA and necessitating islet-cell autoantibody screening demands a clinical approach. Nevertheless, dependable risk assessments based on clinical indicators are currently lacking, and many healthcare providers primarily rely on age and Body Mass Index (BMI) to form their presumptions regarding autoimmune diabetes. Healthcare workers in nations with limited resources are dependent on this clinical supposition. Nothing detrimental has occurred [28].

Clinical features

LADA's clinical, metabolic, and presentations share standard presenting features of T2DM and T1DM as part of a spectrum of varying immunological dysfunctions [29]. LADA-diagnosed patients exhibit less metabolic syndrome and its accompanying variables, such as blood pressure, lipid profiles, and waist-hip ratio, than patients diagnosed with T2DM [30]. Metabolic syndrome is far more prominent in this kind of diabetes than in typical T1DM [31]. It is also seen that there is an increase in the triglyceride levels in LADA-diagnosed patients than in T2DM [32]. Differences between the clinical manifestations of LADA and T2DM have been depicted in a tabular form in Table 1. Differences between the clinical manifestations of LADA and T1DM have been depicted in a tabular form in Table 2.

**Table 1:** Distinguishing features between LADA and T2DM LADA - Latent Autoimmune Diabetes in Adults, T2DM - Type 2 Diabetes Mellitus, HLA - Human Leukocyte Antigen

CLINICAL AND GENETIC FEATURES	LADA	T2DM
Age at which diagnosed	Usually >30-35 years	Any
Resistance for insulin	Usually high	Usually high
β-Cell function	Function is decreased	It might be normal or slightly increased
Insulin requirement	Required usually after 6 months after diagnosis	Absent or might be required years after diagnosis
Body mass index	Normal Rarely obese	>25.0 to >30.0 Overweight or obese
Risk for complications	Increased risk for cardiovascular diseases	Increased risk for cardiovascular diseases
Family history	It may or may not be present	Frequently present
HLA susceptibility	Higher	Slightly increased
Autoimmunity	Slightly raised	Absent

**Table 2:** Clinical features comparison between LADA and T1DM LADA - Latent Autoimmune Diabetes in Adults, T2DM - Type 2 Diabetes Mellitus, HLA - Human Leukocyte Antigen

CLINICAL AND GENETIC FEATURES	LADA	T1DM
Age at which diagnosed	Usually >30-35 years	Seen usually in childhood and very rarely in adulthood
Resistance for insulin	Usually high	Absent
β-Cell function	Function is decreased	Loss of function is seen
Insulin requirement	Required usually after 6 months after diagnosis	Required at the time of diagnosis
Body mass index	Normal Rarely obese	Normal or might be underweight (<18.5)
Risk for complications	Increased risk for cardiovascular diseases	Increased risk for cardiovascular diseases
Family history	It may or may not be present	It may or may not be present
HLA susceptibility	Higher	Increased
Autoimmunity	Slightly raised	Increased

Complication

In instances of undiagnosed LADA or patients erroneously diagnosed as having T2DM and not receiving appropriate treatment, the potential for complications arises. These complications may encompass both microvascular and macrovascular issues. Limited research has been undertaken to investigate the potential microvascular consequences (such as nephropathy, retinopathy, and neuropathy) in individuals with LADA. The existing studies have yielded varied results, partly attributable to significant variations in the duration of illness among the research subjects. The same study revealed that individuals testing positive for GADA exhibited a reduced incidence of microalbuminuria over approximately four to five years [10].

One could posit that individuals with LADA might have a decreased risk of macrovascular complications, such as coronary heart disease, stroke, and peripheral artery disease, owing to their improved metabolic profiles compared to those with T2DM. Nevertheless, recent research has indicated that T2DM and LADA exhibit similar cardiovascular outcomes. No statistically significant disparities were observed regarding cardiac and vascular conditions with LADA and those with T2DM [33].

Treatment

Intervention studies have demonstrated that lifestyle altering can prevent type 2 diabetes. In the Finnish diabetes prevention study and the United States Diabetes Prevention Program (USDPP), dietary changes led to a 59% risk decrease among those with pre-diabetes. Due to a partially comparable aetiology, it is feasible to avoid or delay LADA by making the same changes to one's lifestyle [34]. The discovery of GADA in LADA patients years before diagnosis shows that, like type 2 diabetes, LADA has a protracted pre-diabetic period during which intervening may be viable. In contrast to T2DM, there is intransigence to hormone insulin and this leads to the buildup of disease, LADA is anticipated to have a lower potential for prevention [35].

Although there are recognised criteria for treating classical T1DM, there are very little data addressing LADA medication and no standardised management approach. The treatment strategy used to date for LADA patients varies from clinician to clinician. Furthermore due to camouflaged presenting features, these individuals are rather provided medications for T2DM-specific treatments, which might exacerbate the immunological process and this causes cell death, hastening the path toward insulin dependence, in individuals with increased GADA levels who have traits more like T1DM than T2DM [36].

Insulin therapy

Negligible levels of C-peptide were observed, and insulin therapy is crucial and the sole tool for T1DM patients; persons with LADA advance towards absolute insulin dependence more slowly. Therefore, the critical question is whether insulin therapy is recommended as the first line of treatment for LADA patients. Most research concurs that insulin therapy is safe and efficacious for LADA patients with remaining-cell function [37]. Preclinical research findings indicate that exogenous insulin administration improves cell role and lessens inflammation severity due to decreased glucotoxicity and suppression of islet-cell activity [38].

Sulphonylurea

In a short randomised clinical study, insulin treatment was contrasted with glibenclamide therapy in LADA patients. The study took place in Japan. After 29-32 months of follow-up, patients using sulphonylurea showed poor metabolic control and progressive deterioration of β cell activity, as indicated by a substantial drop in stimulated C-peptide ratio [39]. The least compelling findings in the field have undoubtedly come from studies utilising sulfonylureas. The findings demonstrated that the insulin group's pace of development to an insulin-dependent state was slower than the sulfonylureas group's. Additionally, insulin-treated patients had superior C-peptide preservation than sulphonylurea-treated patients. Furthermore, both research showed improvement of autoantibodies [40].

Dipeptidyl peptidase IV inhibitors

Dipeptidyl peptidase-4 (DPP-4) inhibitors have exceptional safety and efficacy characteristics and are commonly employed to treat patients suffering from chronic kidney disease and diabetes mellitus as a comorbidity. According to a small body of data, much of which is based on animal models, DPP-4 inhibitors may enhance beta cell activity and decrease autoimmune illness. It is because DPP-4 also goes by CD26, a lymphocyte cell surface protein essential for T-cell immunity. It has been demonstrated that DPP-4 activity is connected with waist size, low-density lipoprotein (LDL) cholesterol, hemoglobin A1C (HbA1C) levels, and insulin dose and is higher in persons with LADA than individuals with T1DM and T2DM [41]. A systematic review in China found that sitagliptin combined with insulin glargine preserved C-peptide better than insulin alone over a year in individuals with LADA [42].

Insulin sensitizers

Before the identification of LADA, the majority are initially diagnosed with T2DM and are commenced on metformin treatment. The expert panel concluded that there is no substantiated opposition to utilising metformin; limited data suggests its efficacy in long-term glycemic management. Additionally, it may lead to reductions in body weight, levels of LDL cholesterol, and the likelihood of atherosclerosis development [43].

## Conclusions

A distinct type of diabetes known as LADA combines elements of both T1DM and T2DM. Autoantibodies, which target pancreatic cells that make insulin, are its defining feature. Even while it could start moderate or asymptomatic, it might eventually worsen to the point where insulin medication is necessary. Diagnosis and adequate care are recommended to avoid problems and control ideal blood sugar. More study is required to comprehend the underlying mechanisms further and create tailored treatments for this illness.
